# Early interventional embolization in the treatment of cerebral aneurysm rupture

**DOI:** 10.12669/pjms.346.15786

**Published:** 2018

**Authors:** Song Jiang, Xiufen Xie

**Affiliations:** 1 *Song Jiang, Department Interventional Treatment, Shandong Provincial Qianfoshan Hospital, Shandong University, Jinan, Shandong, 250014, China*; 2 *Xiufen Xie, Department Interventional Treatment, Shandong Provincial Qianfoshan Hospital, Shandong University, Jinan, Shandong, 250014, China.*

**Keywords:** Interventional embolization, Cerebral aneurysm rupture, Clinical effect, Safety

## Abstract

**Objective:**

To analyze the clinical effectiveness and safety of early interventional embolization in the treatment of ruptured cerebral aneurysm.

**Methods:**

Eighty-eight patients with cerebral aneurysm rupture who were admitted to the hospital between February 2015 and October 2016 were selected as the research subjects and were randomly divided into a control group (N=44) and an observation group (N=44) using random number table. Patients in the control group were given interventional embolization three days after admission, while patients in the observation group were given interventional embolization within three days after admission. The complete, sub complete and incomplete embolization rates were compared between the two groups. The prognosis of the patients was evaluated using modified Rankin scale and modified Barthel index. The incidences of complications were recorded.

**Results:**

The complete, sub-complete and incomplete embolization rates of the observation group and control group were significantly different (P<0.05). The modified Rankin score of the observation group was remarkably lower than that of the control group, and the modified Barthel index of the observation group was remarkably higher than that of the control group; the differences had statistical significance (P<0.05). The incidence of complications of the observation group was lower than that of the control group, and the difference had statistical significance (P<0.05).

**Conclusion:**

Early interventional embolization has satisfactory effect in the treatment of cerebral aneurysm rupture and effectively improve prognosis; hence it is worth promotion in clinical practice.

## INTRODUCTION

Cerebral aneurysm refers to nodular protrusion on arterial wall induced by excessive expansion of cerebral arterial cavity. The main cause of cerebral aneurysm is different degrees of congenital defects on local positions of cerebral arterial walls. Nodular bulging which is induced by pressure increase in cerebral arterial cavity is the major independent risk factor for diseases such as subarachnoid hemorrhage.^1^,^2^ A clinical study suggested that cerebral aneurysm rupture mainly manifested as severe headache,^3^ frequent emesis, and disturbance of consciousness, pain of eyepit, nuchal rigidity and coma and had high disability and fatality rates. Another study pointed out that the disability rate and fatality rate of cerebral aneurysm rupture were 50% and 30~40% respectively.^4^

Surgical clipping is a precise method for treating cerebral aneurysm rupture. But with the progress of medicine, interventional embolization has been a frequently used and important therapy in the treatment of cerebral aneurysm rupture.^5^,^6^ Interventional embolization has been recognized by many medical workers and patients as the elimination of craniotomy can reduce damages to patients and accelerate recovery.^7^ However, opinions are different on the implementation opportunity of interventional embolization.^8^ This study investigated the clinical effect and safety of early interventional embolization by carrying out clinical randomized controlled trials on 88 patients with cerebral aneurysm rupture, aiming to provide a reference for the improvement of clinical treatment level.

## METHODS

Eighty-eight patients who were admitted to the hospital between February 2015 and October 2016 and were consistent with the diagnostic criteria of cerebral aneurysm rupture (WTO) were selected as research subjects.^9^ The diagnosis was confirmed by Digital Subtraction Angiography (DSA). Patients who had malignant tumors, other cerebral diseases, intellectual deficiency or mental diseases were excluded. They were divided into an observation group and a control group using random number table. In the observation group (N=44), there were 27 males and 17 females; they aged 23~72 years old (average (48.75±3.22) years old); there were 40 cases of anterior circulation and four cases of posterior circulation; as to Hunt-Hess grading, there were 15 cases of grade I, 14 cases of grade II, 9 cases of grade III, and 6 cases of grade IV; the diameter of aneurysm of 18 patients was smaller than 5 mm, that of 23 patients was between 5 mm and 15 mm, and that of three patients was larger than 15~25 mm. The differences of the general data between the two groups had no statistical significance (P>0.05); hence the results were comparable. All the patients were willing to join in the study and signed informed consent. The study was approved by the ethics committee of the hospital.

### Treatment method

### Surgical method

Interventional treatment was performed on the patients in the observation group in the form of coil embolization within three days after admission, while patients in the control group were given in three days after admission. Except the treatment time, other treatment details were the same in the two groups. The specific treatment process was as follows. Computed tomography angiography (CTA) or DSA was performed before surgery. Moreover relevant treatment was carried out to reduce intracranial pressure, control blood pressure, and prevent upper gastrointestinal hemorrhage and vasospasm. A certain amount of nimodipine injection (Yangtze River pharmaceutical group, China; batch no.: 1201006) was pumped using an intravenous micro-pump; the dose and injection speed were determined by surgeons. General anesthesia was performed after tracheal intubation; arteria femoralis was punctured using Deldinger technology. DSA was performed on bilateral internal carotid and vertebral artery in Towne’s view and conventional lateral view to detect aneurysm and its diameter. Guiding catheter was altered according to the condition of aneurysm, and moreover systemic heparinization was performed. Microguide wire was inserted to aneurysm via the catheter; micro coil in a proper size was used to implement embolization. If there was no retention of contrast agent in the aneurysm after embolization was checked, the micro catheter and guide catheter were removed. The sheath was removed six hour after surgery. The arteria femoralis was compressed at the site where was one cm above the puncture site for 15 min. Conventional pressure dressing was done if there was no hemorrhage. The lower limbs were strictly broken within one hour after surgery. They orally administrated nimodipine tablets (Harbin Pharmaceutical Group, batch no. 1201101) after surgery, three or four times a day.

### Intraoperative nursing

Abnormalities during surgery should be reported to the attending doctor immediately to improve success rate of surgery. Intraoperative nursing included preparation of patients, preparation of articles, intraoperative coordination and prevention of common complications. After the information of the patients was checked, they lay on the back and were continuously given oxygen on an operation table. The puncture site was fully exposed and then disinfected. Articles such as disposable operation kits, catheters for radiography, ordinary catheters and guide wires and drugs such as nimodipine and glycerin fructose were prepared. As to intraoperative coordination, nursing staffs paid close attentions to the disease conditions of patients and implemented corrected nursing modes to observe the changes of disease conditions especially blood pressure and heart rate. Any special condition should be reported to doctors; as a result, doctors could rapidly make predictive processing to prevent adverse reactions and complications and improve success rate of surgery. Moreover infusion tubes were kept smooth; the pressure infusion of artery was closely observed; heparin was supplemented if necessary. The last one was the prevention of complications. The changes of heart rate and blood pressure were paid special attentions to during surgery; blood pressure was measured for 3~5 minutes. The injection of nimodipine was controlled less than 20 mm Hg by anesthetists if there was some problem. The starting time, interval time and supplementary amount of heparin were accurately recorded during surgery. The time of protease activation was measured every hour before and during surgery. Heparin was supplemented to control the time of protease activation at 250~300s or 2.5 times that of before surgery if necessary.

### Observation indexes

The angiography results including complete embolization (100% embolization), sub-complete embolization (90%~99% aneurysm embolization) and incomplete embolization (aneurysm embolization lower than 90%) were observed. Aneurysm rupture, subarachnoid hemorrhage, hydrocephalus, cerebral angiospasm and recurrence were observed after surgery. The patients were followed up. Angiography was performed in the 6^th^ month after surgery. The prognosis was evaluated using modified Rankin scale (mRs) and modified Barthel index (MBI).^10^,^11^ As to mRs, 0~2 points meant favorable prognosis, 0 point meant no symptoms, and six points meant death. As to modified Barthel index, there were 11 events including eating, bath, personal hygiene, dressing, etc; each event was scored as 1~5 points, and higher score indicated stronger living ability.

### Statistical analysis

All the data were statistically analyzed using SPSS ver. 21.0. Measurement data were expressed as mean ± standard deviation (SD). Measurement data such as MBI and mRS were compared using independent sample t test. Enumeration data were expressed by percentage (%). Enumeration data such as embolization effect and incidence of complications were compared using Chi-square test. Difference was considered as statistically significant if P<0.05.

## RESULTS

### Comparison of clinical effect between the two groups

The clinical effect of the observation group was significantly superior to that of the control group according to the postopertative angiography results, and the difference had statistical significance (P<0.05) (Table-I).

**Table-I:** Comparison of clinical effect between the two groups [N(%)]

Group	Complete embolization	Sub-complete embolization	Incomplete embolization
Observation group	37(84.09)	6(13.64)	1(2.27)
Control group	23(52.27)	14(31.82)	7(15.91)
X^2^	12.657	5.359	6.028
P	<0.05	<0.05	<0.05

### Comparison of MRI and mRS between the two groups in the 6th months after surgery

The MRI of the observation group was remarkably higher than that of the control group, and the mRS of the observation group was notably lower than that of the control group, and the differences had statistical significance (P<0.05) (Table-II).

**Table-II:** Comparison of MRI and mRS between the two groups in the 6th month after surgery (mean±SD, point).

Group	MBI	mRS
Observation group	98.58±1.26	1.16±0.27
Control group	80.34±2.57	2.58±0.65
t	25.762	13.138
P	<0.05	<0.05

### Comparison of postoperative complications between the two groups

The incidences of postoperative complications such as hydrocephalus, subarachnoid hemorrhage, aneurysm rupture, cerebral angiospasm and recurrence of the observation group were significantly lower than those of the control group, and the differences were statistically significant (P<0.05) (Table-III).

**Table-III:** Comparison of incidences of postoperative complications between the two groups [N(%)].

Group	Hydrocephalus	Cerebral angiospasm	Aneurysm rupture	Subarachnoid hemorrhage	Recurrence
Observation group	1(2.27)	1(2.27)	1(2.27)	2(4.55)	1(2.27)
Control group	7(15.91)	8(18.18)	7(15.91)	10(22.73)	8(18.18)
X^2^	4.931	7.178	4.931	6.091	7.178
P	<0.05	<0.05	<0.05	<0.05	<0.05

## DISCUSSION

Cerebral aneurysm has many hazards. Delayed treatment may result in high risks of disability and death.^12^ Surgical clipping is easy to cause large trauma to patients previously and has complex operation; moreover multiple complications appear after surgery. Aneurysm which locates deeply has large treatment difficulty and surgical risks.^13^ With the progress of medical technology, interventional embolization has been gradually used to treat cerebral aneurysm. More than 90% of patients with cerebral aneurysm can be treated by interventional embolization and suggest favorable response.^14^ The clinical prognosis of patients with intracranial aneurysm rupture who undergo intravascular interventional treatment was significantly superior to that of patients who undergo microsurgical craniotomy clipping.^15^ But the treatment opportunity of interventional embolization is controversial. It has been pointed out that the presence of blood clot and cerebral tissue swelling could affect the expose of aneurysm of some patients with aneurysm rupture, which could increase surgical risks and difficulties, and that symptomatic treatment such as reducing intracranial pressure and controlling disease condition before surgery and performing surgery after peak stage of occurrence of cerebral angiospasm are helpful to patients.^16^ Performing surgery as soon as possible is also advocated to avoid the deterioration of disease condition.^17^ In this study, the rates of complete embolization, sub-complete embolization and incomplete embilization in the observation group were superior to that in the control group, indicating that early interventional embolization could significantly improve the success rate of embolization for patients with aneurysm rupture, which was consistent with the research results of Hai HY.^18^ Early interventional embolization aims at blocking tumor cavity as early as possible and keeping cavity of aneurysma out of blood circulation of body. For patients with aneurysm rupture, fourteen days after attack is the peak period of aneurysm rupture; vasospasm is of high risks three days after attack; the incidence of vasospasm is the highest seven days after attack.^19^ Vasospasm can affect catheterization and determination on the size of aneurysma. Six months of follow up suggested that the MBI and mRS of the observation group were superior to those of the control group, indicating that early interventional embolization could remarkably improve the recent neurological function and ability of daily living of patients with aneurysma rupture, which was similar to the research results of Liu G et al.^20^ It might be because the occurrence of complications such as hydrocephalus and cerebral angiospasm three days after attack resulted in high risks of rehaemorrhagia and extremely poor prognosis. Interventional embolization treatment at that moment could not reduce the total death rate of patients with aneurysma rupture.

The incidences of complications including hydrocephalus, vasospasm, subarachnoid hemorrhage and aneurysm rupture of the observation group were lower than those of the control group, and the differences were statistically significant (P<0.05), which was similar to the research results of Xue MJ. It indicated that early interventional surgery had high safety. Early interventional embolization in combination with skilled operation of doctors will not aggravate vasospasm and has favorable embolization effect, which can help control disease conditions and avoid further deterioration of disease condition.

## CONCLUSION

Early interventional embolization has remarkable effect in the treatment of cerebral aneurysm rupture as it improves the recent neurological function and ability of daily living and reduced incidences of complications and recurrence rate. However, further studies with longer follow-up time and larger sample size are needed for investigating the long-term prognosis.

### Authors’ Contribution

**SJ & XFX:** Study design, data collection and analysis, Manuscript preparation, drafting and revising.

**XFX:** Review and final approval of manuscript.
